# Febuxostat-induced agranulocytosis in a pediatric hematopoietic stem cell transplant recipient: Case Report and literature review

**DOI:** 10.3389/fphar.2024.1478381

**Published:** 2024-10-23

**Authors:** Debora Curci, Stefania Braidotti, Natalia Maximova

**Affiliations:** ^1^ Laboratory of Advanced Translational Diagnostics, Institute for Maternal and Child Health IRCCS “Burlo Garofolo”, Trieste, Italy; ^2^ Department of Pediatrics, Institute for Maternal and Child Health IRCCS “Burlo Garofolo”, Trieste, Italy

**Keywords:** hematopoietic stem cell recipient, pediatric, hyperuricemia, febuxostat, nonchemotherapy drug-induced agranulocytosis

## Abstract

This report describes a pediatric case of isolated agranulocytosis occurring months after hematopoietic stem cell transplantation (HSCT). Secondary cytopenia, or secondary transplant failure, affects 10%–25% of HSCT recipients, with potential triggers including viral infection, graft-versus-host disease (GVHD), sepsis, and certain medications. Viral reactivation was ruled out based on negative PCR results, while GVHD and sepsis were ruled out based on the patient’s clinical presentation. The patient, who received an HLA 10/10 unrelated donor T-cell transplant, underwent standard myeloablative conditioning to minimize the risk of graft rejection. However, agranulocytosis persisted even after discontinuation of myelotoxic drugs such as valganciclovir and ruxolitinib. Further investigation revealed that the patient had been taking febuxostat, which was subsequently discontinued, leading to a recovery of the neutrophil count. The European Medicines Agency lists agranulocytosis as a rare side effect of febuxostat. The effect of candidate genes and variants involved in febuxostat pharmacokinetics and pharmacodynamics was done using the Pharmacogenomics Knowledge Base (PharmGKB) to accurately evaluate an individual’s risk for neutropenia. This case suggests that genetic variants in renal transporters *ABCG2* (exonic non-synonymous variant, rs2231137), *SLC29A1* (rs747199 and rs628031), and *ABCC4* (3′UTR SNP, rs3742106 and rs11568658) may contribute to drug-induced agranulocytosis. This finding underscores the importance of genetic profiling in the management of patients undergoing HSCT to prevent adverse drug reactions.

## 1 Introduction

Asymptomatic hyperuricemia is not uncommon in hematopoietic stem cell transplantation (HSCT) recipients due to the consolidated use of immunosuppressive agents such as calcineurin inhibitors (Ben Salem et al., 2017). Traditional treatment with allopurinol is associated with complications and interactions that could be potentially serious, leading to lower efficacy if used in regular doses (Tayar et al., 2012).

Febuxostat, a xanthine oxidase inhibitor, reduces urate production and, therefore, reduces serum uric acid levels. It achieves this by inhibiting the conversion of hypoxanthine to xanthine and xanthine to uric acid. Unlike allopurinol, febuxostat is a non-purine selective inhibitor of the xanthine oxidase (XO) enzyme in that it works by inhibiting both the oxidized and reduced forms of XO without inhibiting the enzymes involved in purine or pyrimidine metabolism (Bridgeman and Chavez, 2015). Febuxostat lowers uric acid levels more effectively than allopurinol and serves as an alternative for patients who are intolerant to allopurinol (Becker et al., 2005).

Febuxostat undergoes hepatic metabolism in the cytochrome P450 (CYP) enzyme system into acyl-glucuronide metabolites. This metabolism occurs mainly through conjugation via uridine diphosphate glucuronosyltransferase (UGT) enzymes (UGT1A1, UGT1A3, UGT1A9, and UGT2B7), although a small portion is oxidized into active hydroxyl metabolites (67M-1, 67M-2, and 67M-4) by CYP1A2, CYP2C8, and CYP2C9. Drug interactions *in vitro* studies found that febuxostat had no measurable effect on the activities of the CYP1A2, CYP2C9, CYP2C19, and CYP2D6 isoenzymes (Mukoyoshi et al., 2008). Because XO is involved in the metabolism of azathioprine, mercaptopurine, and theophylline, inhibition of this enzyme can lead to toxicity due to increased drug levels. For this reason, febuxostat is not advised in patients treated with these drugs (Ernst and Fravel, 2009).

Febuxostat has not been reported to cause severe complications in pediatric patients, particularly hematological abnormalities. Still, we have reported one case of isolated severe neutropenia associated with the initiation of febuxostat therapy in a pediatric hematopoietic stem cell transplant recipient.

## 2 Case description

A 10-year-old Caucasian girl was admitted to our department with a high fever and petechial rash. One year before, for a history of fever, cytopenia, and hepatosplenomegaly associated with increased triglycerides and ferritin, sequencing analysis of the *UNC13D* gene evidenced a double-heterozygous mutation. Diagnosis of familial hemophagocytic lymphohistiocytosis type 3 (FLH3) was formalized. The patient underwent treatment according to the HLH-94 protocol with dexamethasone, etoposide, and ciclosporin, obtaining remission after 8 weeks of treatment.

Upon admission to our department, a physical examination revealed high fever, petechiae on the upper and lower limbs, and hepatosplenomegaly. The patient was found to have pancytopenia (neutrophils 430/mm^3^, platelets 13,000/mm^3^, and hemoglobin 8.1 g/dL), hyperferritinemia (ferritin 1,115 μg/L), and low natural killer (NK) cell activity. The FLH3 recurrence was confirmed. The patient was started on dexamethasone and ruxolitinib.

It’s known that HSCT is the only established curative treatment for patients with familial, relapsing, or severe and persistent disease (Janka and Lehmberg, 2013). An HLA-matched, AB0 mismatched unrelated donor was found on the international bone marrow donor registry. The conditioning regimen, which included treosulfan 14 g/m^2^/day for 3 days, fludarabine 160 mg/m^2^ total dose, melphalan 140 mg/m^2^, rituximab 375 mg/m^2^, and antithymocyte globulin was started 6 days before HSCT. Ruxolitinib was discontinued on the first day of conditioning because of possible adverse effects on engraftment and a lack of sufficient data on compatibility between ruxolitinib and drugs used during conditioning (Salit, 2022).

On day 0, they were infused with donor graft containing 11 × 10^6^ CD34+/kg recipient body weight. The prophylaxis of graft-versus-host disease (GVHD) included tacrolimus and mycophenolate mofetil (MMF). Engraftment was achieved on day +11, and complete donor chimerism was reached on day +32. The early post-transplant period was complicated by severe cytokine release syndrome (CRS), which led to acute respiratory failure requiring continuous positive airway pressure (CPAP) and pulmonary hypertension. The IL-6 plasma levels reached 202.8 pg/mL (normal range ≤6.4 pg/mL). The CRS was treated with cortisone, tocilizumab, and continuous infusion of anakinra. Furthermore, a very high type 1 interferon signature (30.9, normal range ≤2.2) led us to restart treatment with ruxolitinib, which replaced the MMF. Furthermore, a high dose of labetalol has been used to correct severe refractory arterial hypertension. On day +12, cytomegalovirus (CMV) reactivation with a high blood viral load further complicated the clinical situation. The patient began antiviral treatment with foscarnet, which gradually decreased the viral load. The CMV clearance was obtained after 4 weeks of antiviral therapy. The foscarnet use caused a worsening of renal function, already compromised by prior treatment with cyclosporin. Foscarnet was replaced with valganciclovir under therapeutic drug monitoring (TDM), which was performed twice a week, maintaining the drug’s blood levels at the normal range’s lower-end cutoffs. Despite the change in antiviral therapy, the estimated glomerular filtration rate (eGFR) declined significantly, falling below 60 mL/min/1.73 m^2^. Taper the dose of tacrolimus was considered. Ruxolitinib with a low dose of tacrolimus was successfully used for GVHD prophylaxis.

In the following 2 months, the patient maintained mild renal dysfunction with persistent proteinuria and albuminuria (400 mg/g of creatinine and 130 mg/g of creatinine, respectively), elevated serum (3,790 ng/mL, normal range 1,010–1730 ng/mL) and urinary (2,657 ng/mL, normal range <300 ng/mL) β2-microglobulin, and an eGFR nailed between 75 and 88 mL/min/1.73 m^2^.

Due to the persistence of high serum uric acid levels (>9 mg/dL, normal range 2.5–6.0 mg/dL), the patient began treatment with allopurinol, which was interrupted after 2 weeks due to the appearance of a widespread skin rash. Because of the persistence of hyperuricemia, treatment with febuxostat 80 mg/day began. Serum uric acid levels dropped rapidly, and after the first week of treatment, the patient did not report any side effects from the new medication. Although the therapy was well tolerated, we documented the tendency towards moderate neutropenia (PMN ≤1,000/mm^3^). A complete blood count repeated 1 week later showed absolute agranulocytosis or grade 4 neutropenia categorized by Common Terminology Criteria for Adverse Events (CTCAE 5.0). No decrease in other blood cell counts was observed. The patient was in excellent condition, with no fever, systemic inflammation symptoms, or laboratory test abnormalities. Treatment with valganciclovir and ruxolitinib was promptly discontinued due to suspected pharmacological myelotoxicity. Furthermore, we tried to stimulate neutrophil production without responding with recombinant human granulocyte stimulating factor (G-CSF; filgrastim). A broad viral work-up was negative for CMV, Epstein-Barr virus (EBV), Adenovirus, Parvovirus, Herpes Simplex virus (HSV), human herpesvirus 6 (HHV-6) and 7 (HHV-7), and hepatitis viruses. The bone marrow smear showed no neutrophils, and the biopsy showed a normal number of megakaryocytes and no evidence of myelodysplasia. Hemophagocytic lymphohistiocytosis infiltration to bone marrow was not present. Peripheral blood full donor chimerism was reconfirmed. Additionally, autoimmune neutropenia (antineutrophil antibody, antinuclear antibody, anti-DNA, and extractable nuclear antigen were negative) and vitamin deficiencies were excluded.

**FIGURE 1:**
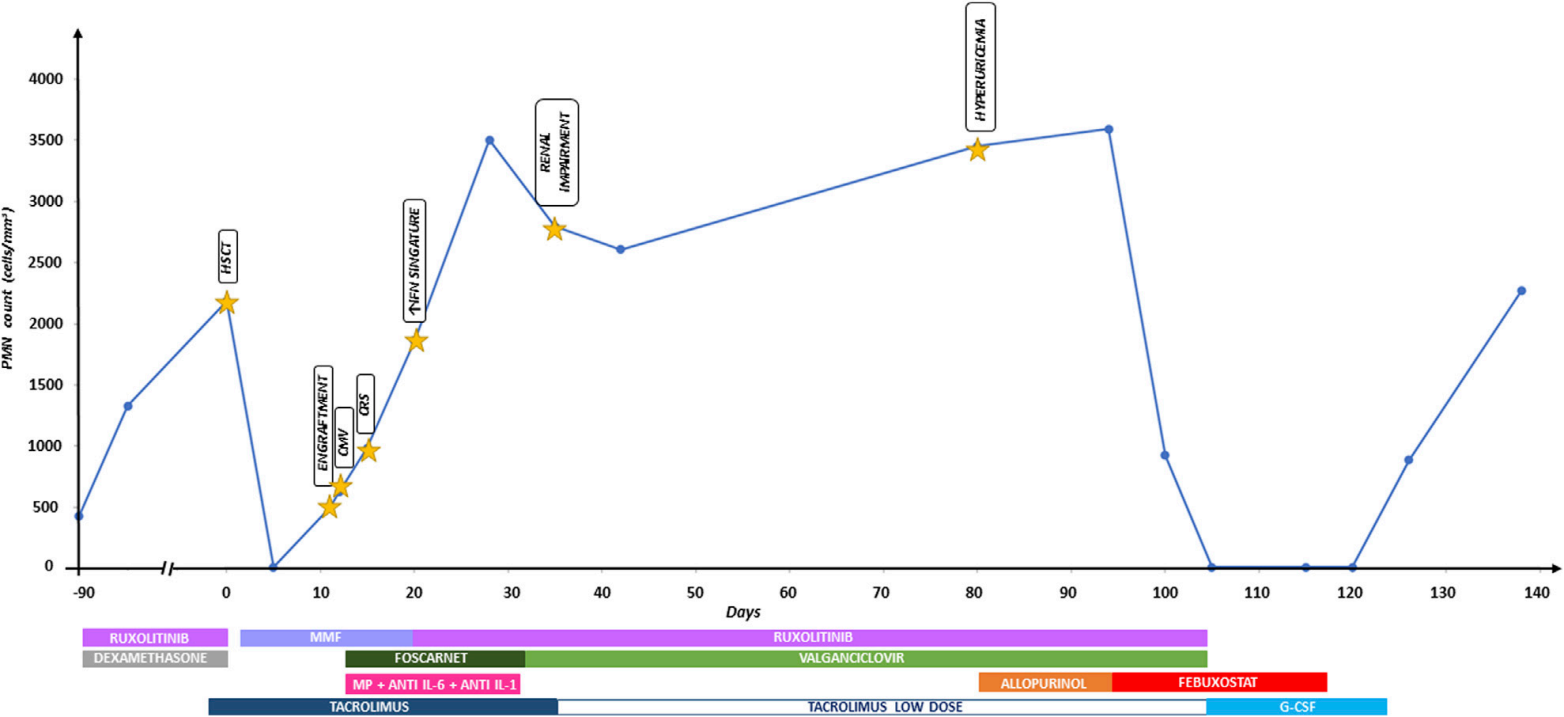
Timeline graph of transplant-related events with corresponding treatments and absolute polymorphonuclear leukocyte (PMN) count trend. Anti IL-6, tocilizumab; anti IL-1, anakinra; CMV, cytomegalovirus; CRS, cytokine release syndrome; G-CSF, granulocyte-colony stimulating factor; HSCT, hematopoietic stem cell transplantation; IFN, interferon; MMF, mycophenolate mofetil; MP, methylprednisolone.

Febuxostat was suspended on suspicion of drug-induced neutropenia. Five days later, the neutrophil count increased to 900/mm3; 2 weeks later, it was normal. Figure 1 shows the timeline graph of transplant-related events with corresponding treatments and absolute polymorphonuclear leukocyte (PMN) count trend.

**FIGURE 2:**
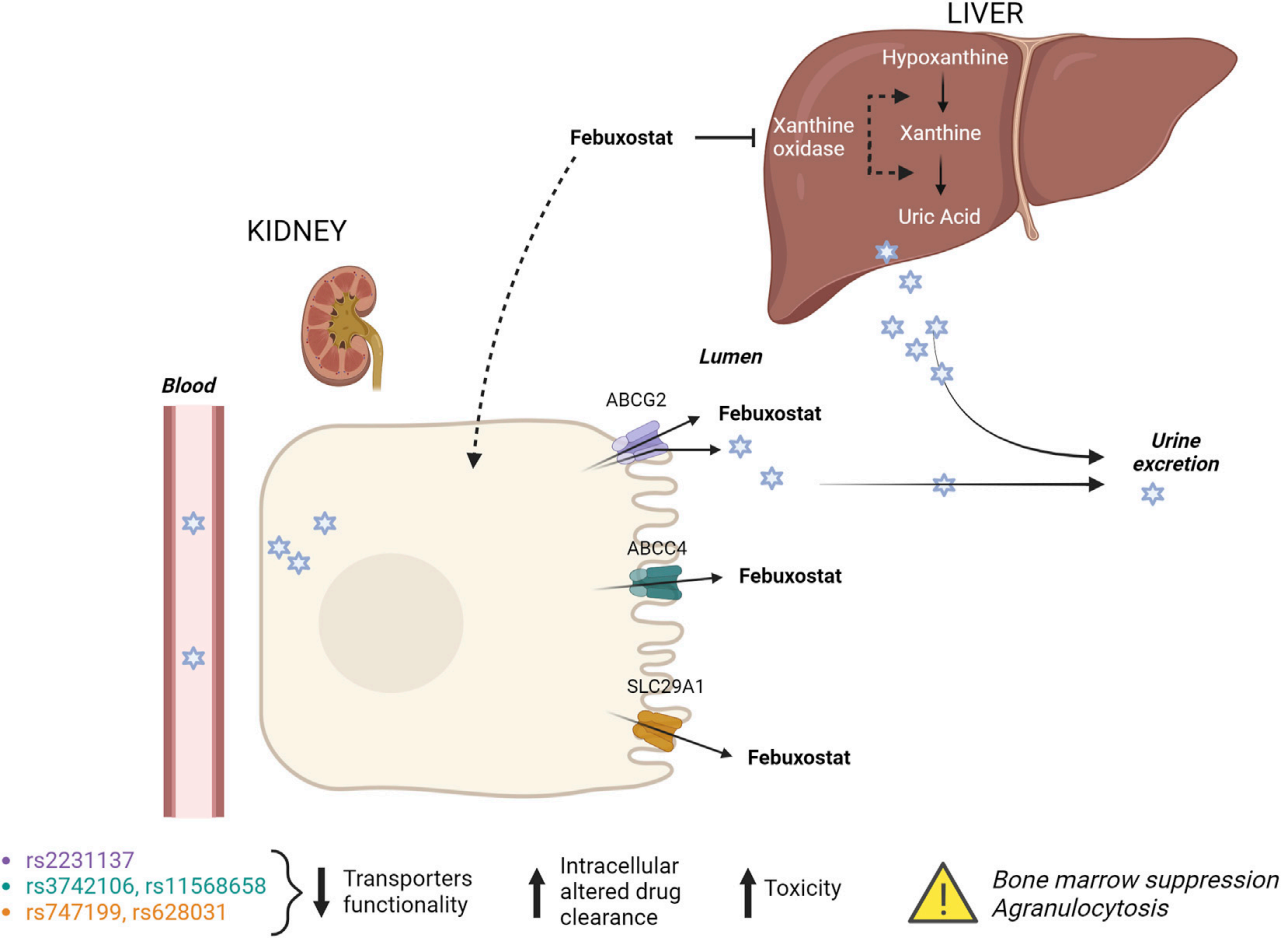
Febuxostat mechanism of action and its interaction with renal transporters. Febuxostat primarily inhibits xanthine oxidase in the liver. Variants in renal transporters may increase the risk of febuxostat-induced agranulocytosis.

The effect of candidate genes and variants involved in febuxostat pharmacokinetics and pharmacodynamics was done using the Pharmacogenomics Knowledge Base (PharmGKB) to evaluate an individual’s risk for neutropenia accurately. In particular, genetic variants in the renal transporters *ABCG2* (exonic non-synonymous variant, rs2231137), *ABCB1* (rs1128503, rs2229109, rs1045642 and rs2032582), ABCC4 (3′ UTR SNP, rs3742106 and rs11568658) and *SLC29A1* (rs747199 and rs628031) was evaluated using Illumina genotyping arrays (Illumina Infinium HumanOmniExpressExome BeadChip). The array includes over 200,000 functional exonic markers, delivering unparalleled coverage of putative functional exonic variants. A total of 200 ng of gDNA (50 ng/μL) for each sample was processed according to Illumina’s Assay protocol. Normalization of raw image intensity data, genotype clustering, and individual sample genotype calls were performed using Illumina’s Genome Studio software. Allele detection and genotype calling were performed with Genome Studio software. The single-nucleotide polymorphism (SNP) of interest and the resulting patient genotypes are reported in Table 1. Figure 2 illustrates the mechanism of action of febuxostat and the drug’s interaction with the mentioned transporters.

**TABLE 1:** Variants associated with febuxostat response according to PharmGKB and genotyping results in the case analyzed by Illumina Infinium HumanOmniExpressExome BeadChip.

	Gene	SNP description	Variant type	Functional effect	Genotype	Phenotype
rs2231137	*ABCG2*	C > T	exonic	reduced efflux capacity of ABCG2 leads to altered drug clearance and increased systemic exposure (risk of agranulocytosis)	CT	heterozygous
rs747199	*SLC29A1*	C > G	intronic	may influence the regulation of SLC29A1; increased risk of neutropenia	GC	heterozygous
rs628031	*SLC29A1*	A > G; A > C	intronic	may influence the regulation of SLC29A1; increased risk of neutropenia	GA	heterozygous
rs3742106	*ABCC4*	A > C	3′-UTR	changes in the regulation of ABCC4 expression leading to an increased risk of agranulocytosis	CA	heterozygous
rs11568658	*ABCC4*	C > A	3′-UTR	changes in the regulation of ABCC4 expression	CC	wild type
rs1128503	*ABCB1*	G > A	exonic	role in drug metabolism and transport	GG	wild type
rs2229109	*ABCB1*	G > A	exonic	role in drug metabolism and transport	GG	wild type
rs1045642	*ABCB1*	C > T; G > A	exonic	role in drug metabolism and transport	GG	wild type
rs2032582	*ABCB1*	C > A/T	exonic	role in drug metabolism and transport	CC	wild type

After 1 year, the patient is doing well, takes no therapy, and leads an normal life. Neutrophil counts were monitored monthly for 6 months after the described episode and were constantly standard. Her kidney function has stabilized, maintaining serum uric acid, proteinuria, and albuminuria values slightly higher than expected, with eGFR returning to levels above 90 mL/min/1.73 m^2^.

## 3 Discussion

In this report, we describe the case of a pediatric recipient who developed isolated agranulocytosis a few months after HSCT.

Secondary cytopenia, also known as secondary graft failure, is a relatively common complication after allogeneic HSCT, characterized by the loss of donor cells after initial engraftment (Bittencourt et al., 2005). From 10% to 25% of HSCT recipients can develop a transitory or permanent decrease in the absolute neutrophil count to less than 500 cells/mm^3^ (Bittencourt et al., 2002). Erythroid and megakaryocytic lineages may also be affected. Conditions associated with an increased occurrence of secondary graft failure including viral infections, especially CMV, HHV-6, and parvovirus, severe, treatment-refractory GVHD, sepsis, HLA disparity, reduced-intensity conditioning, low nucleated cell dose of the graft, T-cell depletion of the graft, allosensitization of the recipient, primary disease recurrence, and use of drugs inducing myelosuppression, such as ganciclovir, trimethoprim-sulfamethoxazole, mycophenolate mofetil (MMF) and ruxolitinib (Salzberger et al., 1997; Bittencourt et al., 2005; Nakamae et al., 2011; Locatelli et al., 2014; Imamura, 2021).

Viral infections account for a large part of post-transplant complications, with up to 90% of patients after allogeneic HSCT likely to experience reactivation of at least one virus (Hill et al., 2017; Düver et al., 2020). Viral infection, in particular CMV, can result in impaired graft function and, in the extreme cases, complete graft failure (Mayer et al., 1997; Dobonici et al., 1998; Steffens et al., 1998; Renzaho et al., 2020). Since viral reactivation could be the most likely cause in this child, we first performed a real-time polymerase chain reaction search for viruses of the Herpesviridae family, Parvovirus, and Adenovirus DNA, which was negative.

Bone marrow is an important target of GVHD, which damages not only stem cells but also the bone marrow niche (Müskens et al., 2021). Bone marrow aspirates from patients with GVHD show reduced numbers of progenitor cells with impaired proliferation in *ex vivo* colony-forming assays compared to patients without GVHD (Martínez-Jaramillo et al., 2001). Sepsis is an extreme example of inadequate host bone marrow response to severe infection. An excessive immune response with initial neutrophilia is followed by profound neutropenia, leukocyte anergy, and, consequently, an inability of the host to control the infection (Zhang et al., 2016).

GVHD was excluded from the causes because the patient had never developed any signs of either acute or chronic GVHD. Sepsis also did not fall within the criteria for differential diagnosis because the patient was in excellent general condition, was asymptomatic, and didn't have inflammation markers.

Other factors that increase the risk of graft failure are HLA-mismatched, T cell-depleted (TCD) or cord blood grafts and non-malignant hematological disease (Beatty et al., 1985; Gluckman et al., 1997; Remberger et al., 2007; Klein et al., 2023). Recipients from unrelated donors have a higher incidence of graft failure than recipients from HLA-identical donors. Comparing unrelated donors, HLA class I disparity was associated with an increased risk of graft failure (Mattsson et al., 2008). When total T-cell depletion was utilized to overcome the HLA barriers in haploidentical HSCT, there was a high rate of disease relapse and graft rejection. Studies of TCD graft rejection demonstrated that residual host-derived cytotoxic lymphocytes were activated and expanded *in vivo*, leading to donor graft rejection by targeting donor mismatched HLA molecules (Bierer et al., 1988). Similar results were obtained from the Spanish Group for Allogeneic Peripheral Blood Transplantation in CD34^+^ selected HSCT from HLA-identical siblings (Urbano-Ispizua et al., 2001). This study shows that the number of CD3+ cells in the graft with a threshold of 0.2 × 10^6^/kg or less is the most critical factor in maintaining sustained engraftment in CD34+ selected HSCT from HLA-identical siblings.

Having received the T-cell repleted graft, which contained a very high number of stem cells, from an HLA 10/10 matched unrelated donor (MUD), our patient did not fall into the high-risk category for these factors.

Due to the low number of hematopoietic cells in a single harvest, allogeneic cord blood transplants have been employed mainly in treating children needing HSCT. However, in almost all published studies, the most important and limiting factor influencing cord blood transplant outcome resulted from the low cell dose infused, which was found to correlate with the rate of engraftment, speed of hematopoietic recovery, frequency of graft failure, and survival (Rubinstein et al., 1998; Rocha et al., 2001; Eapen et al., 2007; Kurtzberg et al., 2008). Allosensitization towards major HLA antigens or, less frequently, minor histocompatibility antigens that develop the heavily transfused recipients can contribute to the increased rejection rate in non-malignant diseases. In aplastic anemia, multiply transfused patients are more likely to develop graft rejection. Thus, HSCT performed at an early age is highly recommended to limit sensitization to histocompatibility antigens. As a consequence of disease status and numerous pretransplant transfusions, in analogy with aplastic anemia, the incidence of graft failure in thalassemia ranges from 8% to 12% according to the patient’s risk class (Bacigalupo et al., 2010). Our patient received only a few packed red blood cells and platelet units at the onset and the recurrence of hemophagocytic lymphohistiocytosis. Therefore, she wouldn't be at risk of allosensitization due to transfusions.

Other important factors for developing secondary graft failure are non-myeloablative (NMA) or reduced-intensity conditioning (RIC) and low-intensity pre-transplant immunosuppression. High-dose myeloablative radio and chemotherapy (MAC) are conventionally used as conditioning for HSCT, with advantages in transplant-related outcomes. MAC has a profound immunosuppressive effect on the host, limiting the ability to reject the graft (Petersen, 2007). RIC or NMA HSCT resulted in a three to four times increased risk of graft failure compared with MAC transplant (Olsson et al., 2013). Even an alloreactive immunological reaction mediated by residual host immunity persisting after the conditioning regimen can induce graft failure. Residual host T-cells are the most prominent effector cells mediating rejection (Masouridi-Levrat et al., 2016). T-cell-mediated graft rejection can occur in both HLA-mismatched and HLA-matched settings. In the latter case, rejection is due to responses directed against minor histocompatibility antigens (Kernan et al., 1987; Voogt et al., 1990).

Our patient received a standard myeloablative conditioning regimen associated with rabbit antithymocyte globulin (Thymoglobulin) to minimize the possibility of rejection.

Determining post-transplant chimerism is an essential aspect of post-transplant follow-up. It allows us to distinguish between graft failure, poor graft function, or primary disease recurrence (Delie et al., 2021). The peripheral blood chimerism determination showed that the patient has a stable chimera, confirming full donor chimerism.

Furthermore, a bone marrow biopsy excluded the primary disease recurrence. The hallmark of the FLH bone marrow picture is the presence of prominent hemophagocytosis with a decreased number of normal hematopoietic precursors (Henter et al., 1991). In our case, agranulocytosis was the only pathological finding. The bone marrow smear instead suggested probable drug toxicity. Valganciclovir and ruxolitinib were discontinued at the first evidence of neutropenia, as their myelotoxic effect is well known. Both drugs impair bone marrow cell proliferation, either by inhibiting DNA replication in bone marrow progenitor cells (valganciclovir) or disrupting cytokine-mediated survival signals (ruxolitinib), resulting in significant myelotoxicity (Salzberger et al., 1997; Locatelli et al., 2024). Even after 10 days of myelotoxic drug interruption and continuous G-CSF stimulation, agranulocytosis persisted.

The patient was still taking febuxostat, which replaced allopurinol due to its known side effects. Febuxostat therapy was discontinued, resulting in a rise in neutrophil count after 5 days.

The European Medicines Agency patient information leaflets on febuxostat report agranulocytosis as a rare event collected in the post-marketing experience. In the literature, we found only three reports of neutropenia/agranulocytosis following treatment with febuxostat. All three cases involved adult patients with chronic renal failure and other significant comorbidities (Kobayashi et al., 2013; Poh et al., 2017). A later published review reported no association between febuxostat treatment and agranulocytosis was confirmed (Jordan and Gresser, 2018).

The administration of febuxostat as an ABCG2 inhibitor may alter the pharmacokinetics and efficacy of ABCG2 substrate drugs. Patients with *ABCG2* SNPs (decreased ABCG2 function) reportedly exhibit higher bioavailability of *ABCG2* substrates (Yamasaki et al., 2008) than subjects with *ABCG2* wild type (WT). Therefore, febuxostat could induce a similar effect on drug absorption. The genetic variant rs2231137 is known to reduce the efflux capacity of the ABCG2 transporter, leading to increased intracellular concentrations of substrates, among which febuxostat (Yamasaki et al., 2008). Higher intracellular drug concentrations may lead to increased toxicity, potentially affecting the bone marrow and leading to agranulocytosis. In addition, reduced ABCG2 function could lead to febuxostat accumulation in the kidneys, resulting in altered drug clearance and increased systemic exposure, further contributing to the risk of agranulocytosis.

For this reason, the presence of missense SNP in *ABCG2*, concomitant with other variants in renal drug transporters (*SLC29A1* and *ABCC4*) in heterozygous, could affect the clinical drug efficacy in terms of risk of neutropenia. It is known that patients with rs11568658 in *ABCC4* and kidney transplant may have an increased risk of neutropenia when treated with valganciclovir compared to patients with WT (Billat et al., 2016). For this reason, another genetic variant in the 3′ UTR of *ABCC4* (rs3742106) may lead to changes in the regulation of ABCC4 expression, potentially reducing the efflux of febuxostat and its metabolites (Billat et al., 2016). Reduced ABCC4 function can result in higher intracellular concentrations of toxic metabolites, contributing to cellular toxicity in the bone marrow and ultimately leading to agranulocytosis. Additionally, impaired drug efflux could exacerbate febuxostat’s pharmacodynamic effects, increasing the risk of drug-induced bone marrow suppression. The variants rs747199 and rs628031 in *SLC29A1*, involved in the transport of nucleosides and some drugs across cell membranes could also affect the cellular uptake of febuxostat or its regular metabolism, leading to either increased exposure to toxic metabolites or an imbalance in cellular nucleotides, thereby contributing to bone marrow suppression and agranulocytosis. Lee et al. have demonstrated an association between neutropenia and genetic polymorphism in *SLC29A1* (rs747199) in pediatric patients with inflammatory bowel diseases in treatment with thiopurine (Lee et al., 2015). In line with this result, our patient, heterozygous for variants in *SLC29A1* (rs747199 and rs628031), may have developed neutropenia after including febuxostat in the therapeutic protocol.

The identified variants in *ABCG2*, *SLC29A1*, and *ABCC4* may collectively contribute to an increased risk of febuxostat-induced agranulocytosis by altering the drug’s pharmacokinetics and pharmacodynamics through the following possible mechanisms: altering drug transport and clearance inducing higher systemic and intracellular levels of febuxostat; increasing exposure to toxic metabolites, leading to bone marrow suppression and disrupting normal cellular processes, which affect neutrophil production and survival. This case demonstrates that there exists an association between concomitant treatments (valganciclovir, tacrolimus, and febuxostat) and the onset of neutropenia. There are several limitations in this case. First of all, a single case limits the significance of these findings. Moreover, during treatment with febuxostat, the patient received other drugs, such as valganciclovir and ruxolitinib. The potential contributions of these additional drugs as the trigger of agranulocytosis due to febuxostat cannot be ruled out.

In conclusion, this case report suggests that the presence of *ABCG2*-*SLC29A1*-*ABCC4* haplotypes may affect clinical outcomes, leading to neutropenia.

However, it is important to understand that after excluding febuxostat medication, resolution of neutropenia was observed, indicating that the identified haplotype could help clinicians choose the treatment option to avoid undesired effects. Complete blood count close monitoring should be recommended at the beginning of the treatment to monitor for side effects in individuals with renal failure or treatment-related frailty. In hospitals with the necessary tools, genetic tests to identify polymorphisms in candidate genes before starting febuxostat treatment could offer significant value for some patients.

## 4 Patient perspective

The patient’s parents were pleased that the cause of the neutropenia was identified, and the complication resolved relatively quickly without subjecting the daughter to further treatments.

After stopping all medicine, they were grateful for the improvement in their daughter’s quality of life.

## Data Availability

The datasets presented in this study can be found in online repositories. The names of the repository/repositories and accession number(s) can be found in the article/supplementary material.
